# Pre and Post Synaptic NMDA Effects Targeting Purkinje Cells in the Mouse Cerebellar Cortex

**DOI:** 10.1371/journal.pone.0030180

**Published:** 2012-01-19

**Authors:** Etienne Lonchamp, Frédéric Gambino, Jean Luc Dupont, Frédéric Doussau, Antoine Valera, Bernard Poulain, Jean-Louis Bossu

**Affiliations:** Centre National de la Recherche Scientifique, associé à l'Université de Strasbourg, Institut des Neurosciences Cellulaires et Intégratives, Strasbourg, France; The Research Center of Neurobiology-Neurophysiology of Marseille, France

## Abstract

N-methyl-D-aspartate (NMDA) receptors are associated with many forms of synaptic plasticity. Their expression level and subunit composition undergo developmental changes in several brain regions. In the mouse cerebellum, beside a developmental switch between NR2B and NR2A/C subunits in granule cells, functional postsynaptic NMDA receptors are seen in Purkinje cells of neonate and adult but not juvenile **rat** and mice. A presynaptic effect of NMDA on GABA release by cerebellar interneurons was identified recently. Nevertheless whereas NMDA receptor subunits are detected on parallel fiber terminals, a presynaptic effect of NMDA on spontaneous release of glutamate has not been demonstrated. Using mouse cerebellar cultures and patch-clamp recordings we show that NMDA facilitates glutamate release onto Purkinje cells in young cultures via a presynaptic mechanism, whereas NMDA activates extrasynaptic receptors in Purkinje cells recorded in old cultures. The presynaptic effect of NMDA on glutamate release is also observed in Purkinje cells recorded in acute slices prepared from juvenile but not from adult mice and requires a specific protocol of NMDA application.

## Introduction

NMDA receptors are glutamate activated ion channels that shape the response of postsynaptic neurons and regulate presynaptic neurotransmitter release ([1], [2] for reviews). The postsynaptic effect is mediated by synaptic NMDA receptors localized on dendritic spines and/or by extrasynaptic receptors localized on dendritic shafts and the soma ([3] for a review). The presynaptic effect is mediated either directly by NMDA receptors localized on axons or synaptic boutons, or indirectly by postsynaptic receptors allowing the release of retrograde messengers acting on the presynaptic element ([2], [4] for reviews). The presynaptic effect of NMDA is involved in several forms of short term and long term synaptic plasticity ([5] for a review).

Functional NMDA receptor channels are tetrameric assemblies formed by the association between two NR1 subunits (represented by a family of eight different splice variants) with NR2A-D subunits. NR3A-B subunits have also been identified and are believed to form ternary complexes by co-assembly with NR1 and NR2 subunits. The subunit composition of the NMDA-R determines the NMDA channel biophysical properties, permeability to calcium, sensitivity to Mg^2+^ and pharmacology ([6] for a review). NMDA receptor subunit expression is regulated and the NR2A/NR2B ratio changes with development, sensory experience and synaptic plasticity ([5] for a review).

In the cerebellum, NR1 subunits of NMDA receptors are detected in all neurons of young and adult rodents, whereas the NR2 A/B and NR3B subunits exhibit a distinct pattern of expression depending on the cell type and the degree of development and maturation [7]–[12]. Electrophysiological recordings demonstrate the existence of functional synaptic NMDA receptors in granule cells [9], [13], [14], [15], Golgi cells [16], brush cells [14] and Purkinje cells from adult mice [17], [18] and adult rats [19]. Extrasynaptic NMDA receptors have been characterized on granule cells 0 [20] stellate cells [21] and Golgi cells [16], [22] irrespective of age, while their presence in Purkinje cells has only been detected in adult mice and new born rats [17], [23].

Presynaptic effects of NMDA in the cerebellar circuitry have been extensively studied in the past decade. It is well established that NMDA facilitates presynaptic release of GABA onto Purkinje cells, basket cells and stellate cells [24], [25]. Functional NMDA receptors are present at GABAergic terminals of cultured cerebellar interneurons [26] and on the axonal pinceau of basket cells [8].

Application of NMDA also depresses the parallel fiber-Purkinje cell excitatory postsynaptic current [27], whereas application of NMDA receptor antagonists blocks long term depression (LTD, [28]). More recently, presynaptically expressed long term depression (pre-LTD, [29]) and long term potentiation (pre-LTP, [30]) have been characterized at this synapse. Both pre-LTD and pre-LTP are mediated by NMDA receptor-dependent release of NO and endocannabinoids [29], [30]. The exact localization of NMDA receptors involved in LTP or LTD is still controversial, with two alternatives being proposed: (1) they are localized at interneuron axon terminals, or (2) at parallel fiber terminals ([4], for a review). Arguing against the presence of presynaptic NMDA receptors on the terminals of parallel fibers, it was shown that the frequency of miniature excitatory postsynaptic currents (mEPSCs) recorded in Purkinje was not modulated by NMDA [24], and that NMDA receptors do not contribute to presynaptic calcium transients in parallel fibers during single or repetitive stimulation [30]. Nevertheless the presence of NMDA receptor subunits was detected on parallel fiber terminals [8], [31].

Based on observations showing that the subunit composition of NMDA receptors undergoes several changes during development particularly in the cerebellum, we asked whether the effects of NMDA (pre and /or postsynaptic) at the granule cell-Purkinje cell synapse are regulated during development. Using whole cell recordings of Purkinje cells we investigated the effects of NMDA in mouse cerebellar slices maintained in culture for periods of time between 10 and 40 days. We show that NMDA in the presence of TTX facilitates glutamate release onto Purkinje cells. Nevertheless, we demonstrate that this effect is only detectable in young (17–22 days old) cultures. Furthermore we confirm that old (28–45 day old) Purkinje cells express functional extra-synaptic NMDA receptors. The presynaptic effect of NMDA on glutamate release is also observed in Purkinje cells recorded from acute slices prepared from juvenile mice (but not adult). However, in this preparation several successive or long lasting applications of NMDA are required to elicit such an effect.

Our data demonstrate that the developmental switch between pre and post synaptic location of NMDA receptors targeting at Purkinje cells synapses is observed in slice cultures and acute slices, suggesting that climbing fibers are not required for this switch to occur. The slices cultures would be a good model to examine the effect of trophic factors on the expression of functional NMDA receptors whereas acute slices provide an adequate model to analyze the consequence of the developmentally regulated expression of functional NMDA receptors targeting Purkinje cells on synaptic plasticity.

## Materials and Methods

### Ethic statement

All experiments have been conducted using protocols designed according to the European and French guidelines on animal experimentation and approved by the direction of the Bas-Rhin veterinary office, Strasbourg, France; authorization number 67–295 to JdB), and the direction of Paris veterinary office, France, (authorization number 75–279 to MP).

### Organotypic slice cultures

Organotypic cultures were prepared from cerebella removed from 1–10 day-old mice [15], [32]. The cerebellum was dissected after decapitation and parasagittal cerebellar slices of 400 µm-thickness were cut using a McIlwain tissue chopper. Individual slices were embedded in clotted chicken plasma on glass coverslips and placed in culture tubes containing 750 µl of culture medium made of 25% heat-inactivated horse serum, 50% Eagle's basal medium, 25% Hank's buffered salt solution (HBSS) supplied with 33.3 mM D-glucose and 0.1 mM glutamine. The tubes were placed in a roller drum inside an incubator at 36°C. Uridine (Sigma, Saint Louis, USA), cytosine-β-D-arabino-furanoside (Sigma) and 5-fluorodeoxyuridine (Sigma) were used in combination (10^−7^ M working solution) and added to the culture medium for 24 hours, 2 to 4 days after the culture was started in order to retard the overgrowth of macrophages, glial cells and fibroblasts. The cultures were fed once a week by renewing the culture medium. Electrophysiological recordings were performed after 1–5 weeks in *vitro*. The age of the culture and/or Purkinje cells was calculated as follows: postnatal age of the mouse used for preparing the slice cultures + number of days in culture.

### Acute slices

Standard procedures were used to prepare 300-µm-thick parasagittal slices from mice at postnatal days P14–16 or P40–P50. Briefly, mice were killed by decapitation under isoflurane anaesthesia. Brains were dissected in ice-cold artificial cerebrospinal fluid (ACSF), and sliced with a vibratome (Leica VT1200S) at 4°C. Slices were maintained for 30 min at 32°C in an interface chamber containing ACSF equilibrated with 95% O_2_, 5% CO_2_ and containing (in mm): NaCl 124, KCl 2.7, CaCl_2_ 2, MgCl_2_ 1.3, NaHCO_3_ 26, NaH_2_PO_4_ 0.4, glucose 10, ascorbate 4, then held for at least 1 h at room temperature before being transferred to a superfusing recording chamber.

### Electrophysiological recordings

Cerebellar slices were placed in a recording chamber on the fixed stage of an upright microscope (Nikon). Patch-clamp recordings were carried out under voltage clamp in the whole-cell (WCR) or outside-out recording modes using an Axopatch 200 A amplifier (Axon Instruments, Foster City, CA) at room temperature (22–24°C). Purkinje were identified by their typical morphology: large (15–20 µm) neurons localized at the periphery of the cultures, characterized by highly refringent nucleoli. Electrodes of 5 MΩ were pulled from borosilicate glass capillaries (Clark Electromedical Instruments, Pangbourne, England), and filled with a solution containing in mM: K^+^-gluconate 132, ethylene-glycol-tetraacetic-acid (EGTA)/KOH 1, MgCl_2_ 2, NaCl 2, Hepes/KOH 10, MgATP 2, and GTP 0.5. pH was adjusted to 7.2 with TrisOH. The recorded current was digitized at 20 KHz using a Digidata 1320 (Axon Instruments) and stored on a PC hard disk. Off-line analysis was performed using MiniAnalysis (Synaptosoft Inc, Fort Lee, NJ) and pClamp-8 (Axon Instruments) softwares, prior analysis data were filtered at 1 KHz.

mEPSCs were detected semi-automatically using the Minianalysis software with specificic parameters for EPSC detection. The event-detection threshold was fixed at 5–6 pA, and the area threshold at 20–25 and thereafter held constant. Amplitude and inter-event intervals were determined for recording period of 100 sec. Cumulative inter-event interval distribution was prepared for comparing mEPSC frequency before and after experimental manipulations. The Kolmogorov-Smirnov test was used to determine if the two distributions were different, using a criterion of P>0.05. The mean frequency was determined using the mean of inter-event intervals. The mean amplitude of mEPSCs in each experimental condition was determined and compared using a **Wilcoxon** paired test, for non-normal distribution values using a criterion of P>0.05. In some cells the mEPSC amplitude histogram distribution was constructed, the value of the peak of the amplitude distribution being determined using a fitting procedure.

### Physiological medium and drugs

During the course of the experiments the cultured slices were continuously superfused with a solution containing in mM: NaCl 130, KCl 2.7, CaCl_2_ 5, Hepes/Tris 10, glucose 5.6. pH was adjusted to 7.4 with TrisOH. Acute slices were continuously superfused at 20°C with a solution ACSF equilibrated with 95% O_2_/5% containing in mM: NaCl 124, KCl 2.7, CaCl_2_ 3, NaHCO_3_ 26, NaH_2_PO_4_ 0.4, glucose 10, ascorbate 4, Tetrodotoxin (TTX , 2 .10^−6^ M) and Gabazine (10^−5^ M) were added in the external solution 20 minutes before the recordings.

The concentration of CaCl_2_ was increased to 5 mM (for slice cultures) or 3 mM (for acute slices) in order to compensate for the absence of Mg^2+^.

Stock solutions of (2R)-amino-5-phosphonovaleric acid (AP5, Sigma) 10^−2^ M, bicuculline methiodide (Sigma) 10^−2^ M, Gabazine (Tocris) 10^−2^ M, N-methyl-D-aspartate (NMDA, Sigma) 5×10^−2^ M, MK801 10^−2^ M (Sigma), TTX (Sigma) 2.10^−4^ M, were prepared in distillated water and 6-cyano-7-nitroquinoxaline-2,3-dione (CNQX, Sigma) 10^−2^ M, 2,3-dihydroxy-6-nitro-7-sulfamoyl-benzo-quinoxaline-2,3-dione (NBQX, Sigma) 10^−2^ M in ethanol. When acute applications of the pharmacological agents were needed drugs were diluted from stock solutions in the external solution immediately before the experiments. The time needed to achieve full perfusion within the slice is about 30–60 sec, it is the time required for CNQX to block mEPSC or the time required for TTX to block EPSC.

## Results

In order to examine the pre and post synaptic effects of NMDA on Purkinje cells NMDA was applied in the bath solution in the absence of external Mg^2+^. The response to NMDA was characterized firstly in Purkinje cells recorded from organotypic slice cultures at a holding potential of −40 mV, and secondly in Purkinje cells recorded from acute slices at a holding of −60 mV. In slice cultures, data were expressed as a function of age of Purkinje cells (for the postsynaptic effect) or the culture (for the presynaptic effect), whereas in acute slices the data were reported as a function of the postnatal age of the animal.

Extra synaptic NMDA receptors are expressed in old (more than 28 day old) Purkinje cells recorded from slice cultures.

**Figure 1:**
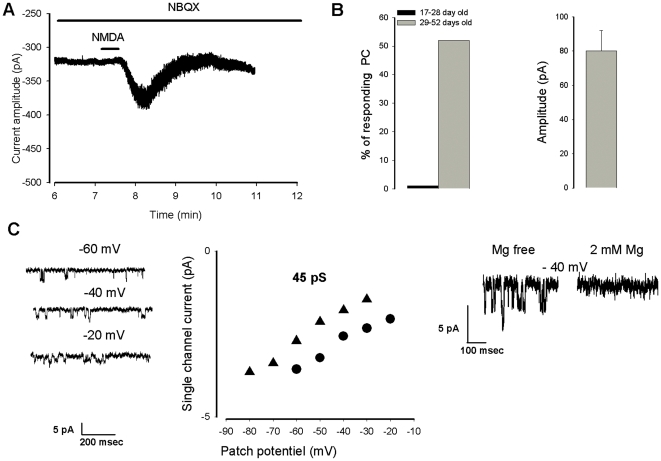
A postsynaptic response to NMDA in old Purkinje cells. A. NMDA-induced inward current recorded in a 45 day old Purkinje cell in the presence of the AMPA receptor antagonist NBQX. B. (left) represents the number of Purkinje cells responding to NMDA with an inward current as a function of their age. B (right) mean amplitude of the NMDA inward current (10^−5^ M, for 30 s). C, single NMDA channel recordings in outside-out patches, left: current traces illustrating the NMDA channels, middle: I/V plots obtained with two outside-out giving a conductance close to 45 pS, right: voltage dependent blockade of the NMDA channels.

In the presence of TTX, application of NMDA 5×10^−5^ M during 30 s induced a long lasting (∼2 minutes, n = 8) inward current in about 50% of Purkinje cells older than 28 days (Fig. 1A and B). The inward current had a mean amplitude of 77±17 pA, n = 8 (Fig. 1B). The NMDA-induced current was still observed in the presence of the specific AMPA receptor antagonist NBQX (see Fig. 1A, n = 4), but was blocked in the presence of 2×10^−3^ M external Mg^2+^ (data not shown, n = 3). The direct evidence of the presence of extra synaptic NMDA receptors in Purkinje cells older than 28 days is illustrated in Fig. 1C. Two outside-out patches were obtained from two Purkinje cells responding to NMDA with an inward current. NMDA was then applied in the bath and after 1–2 minutes single channels with a conductance of 45 pS were detected. The channel activity was blocked by external Mg^2+^.

NMDA induces an increase in the frequency of mEPSCs in 17–22 day old slice cultures.

**Figure 2:**
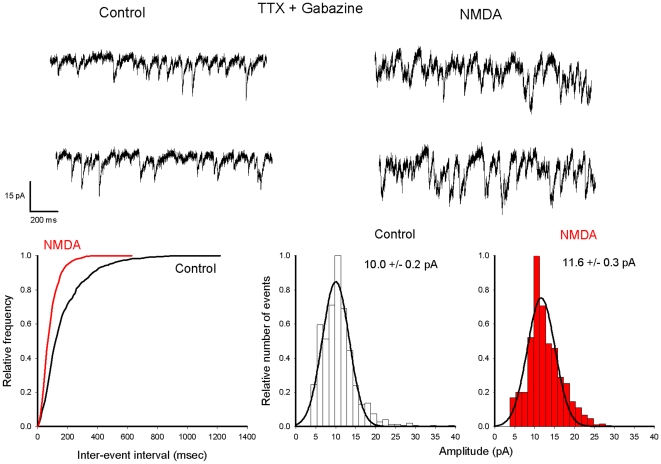
NMDA increases the frequency of mEPSCs in Purkinje cells recorded in a young (P18) slice culture. Upper record: currents traces obtained at −40 mV in the presence of TTX and Gabazine before (left) and after NMDA application (right). Transient inward currents represent mEPSCs. Lower graphs: (same cell as in A) cumulative probability plots of inter-event intervals (left) under control conditions (black trace) and in the presence of NMDA (red trace); amplitude histogram distribution under control conditions (middle) and in the presence of NMDA (right). In both cases the amplitude distribution was fitted using a gaussian function with a peak value indicated on each graph. Note that in the presence of NMDA the distribution of inter-event intervals is shifted towards shorter intervals whereas the amplitude distribution is almost unchanged.

**Figure 3:**
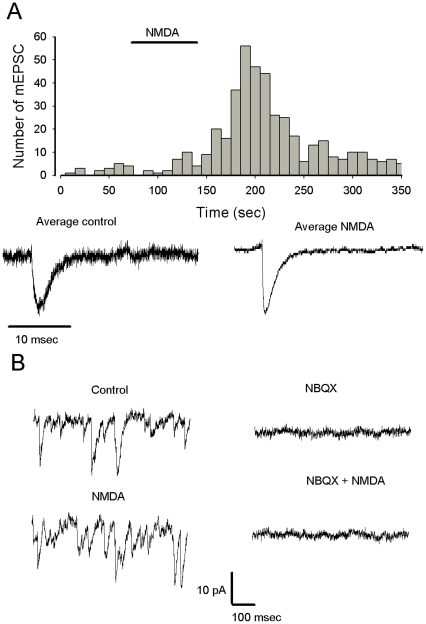
The effect of NMDA is specific for mEPSC. A. Histogram represents the number of mEPSCs as the function of the recording time (bin 10 s), NMDA is applied as indicated by the horizontal bar. Current traces represent average of events selected in control conditions between 0 and 50 sec (right), and at the peak of the increase of frequency induced by NMDA between 180 and 220 sec. Note that the two average currents display the same kinetic properties that fit with mEPSC (the trace on the left is more noisy because the average is constructed with few events). B. Left: mEPSCs recorded in control conditions (top trace) and after NMDA (bottom trace). Traces on the right (same cell), current trace in the presence of NBQX (top trace, mEPSCs are blocked) and after co-application of NMDA (bottom trace, no further effects of NMDA are detected).

**Figure 4:**
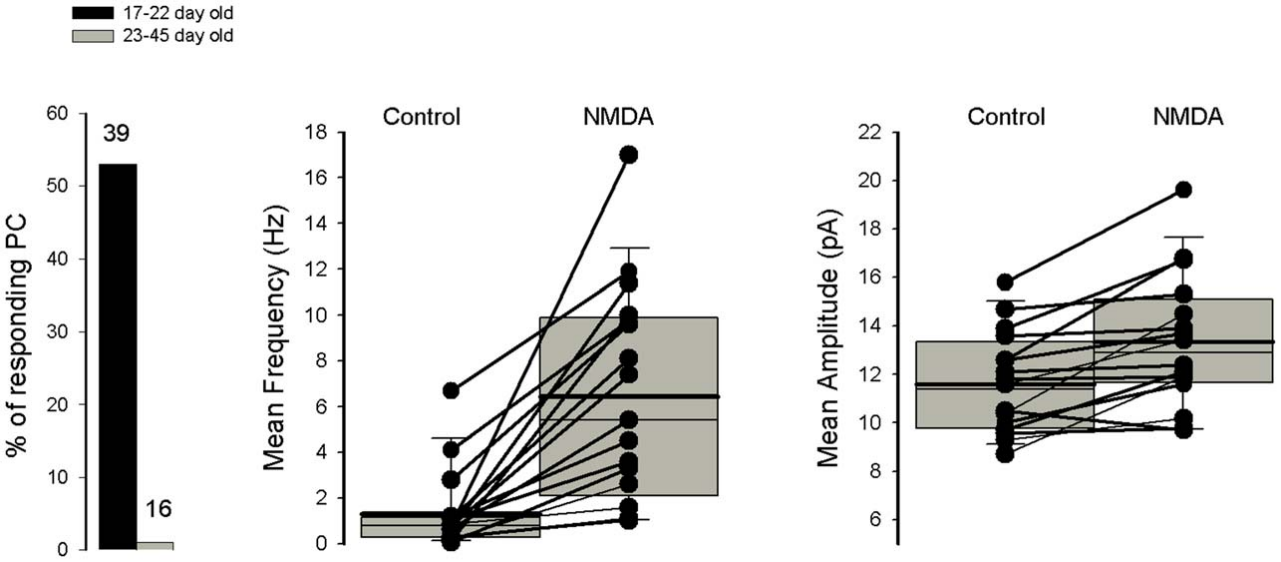
Summary of NMDA effects on mEPSCs recorded in Purkinje cells recorded from slice cultures. Graph on the left represents % Purkinje cells in which an NMDA-mediated increase in mEPSC frequency is observed as a function of Purkinje cell age. Graphs in middle and on the right represent respectively the mEPSC mean frequency and mean amplitude before and after the first application NMDA recorded in Purkinje cells from 12–22 day old slice cultures. A representation with box plots indicating the mean values (thick line) and median values (thin line) has been adopted because of the non-normal distribution of mean frequency and mean amplitude values. The bottom and the top of the box represent the 25^th^ and 75 th percentile respectively. The ends of the whiskers represent the 5^th^/95^th^ percentile.

mEPSCs were recorded in isolation in the presence of TTX and Gabazine, a specific blocker of γ-aminobutyric-acid (GABA)_A_ receptor. NMDA was applied for 60 sec at a concentration of 10^−5^ M. A typical example for a 17–22 day old slice culture is illustrated in Fig. 2. After a delay of 30 sec (see Figure 3, upper part) NMDA induced an increase of mEPSC frequency (compare figure 2 the cumulative distribution of inter-event intervals before and during NMDA, bottom left) without changing the holding current and the peak of the mEPSC amplitude distribution (compare figure 2 the two amplitude distribution histograms, bottom right). When the frequency of mEPSCs was relatively low, with a majority of individual mEPSCs, an average of events detected in control and in presence of NMDA could be compared (Fig. 3A). The average current in both conditions shared the same kinetic properties that are typical for mEPSC. Miniature EPSCs were blocked by the AMPA receptor antagonist NBQX, and in this condition NMDA did not increase current noise or affect the holding current. (Fig. 3B n = 3). Fig. 4 summarizes the data obtained on 55 Purkinje cells. The facilitatory effect of NMDA on AMPA mediated mEPSCs was observed on about 50% of Purkinje cells recorded in 17–22 day old slice cultures, and was never detected in Purkinje cells recorded in older cultures (histogram on the left). In 17 Purkinje cells recorded in 17–22 day old slice cultures the cumulative inter-event interval distribution was significantly different after the first application of 10^−4^ M NMDA, with a reduction of the mean inter-event interval indicating an increased frequency. NMDA increased the mEPSC frequency from 1.3±0.4 Hz to 6.4±1.1 Hz (n = 17, ± SE, p = 0.00012, Fig. 4, middle). Probably because of the presence of overlapping mEPSC, mean amplitude of mEPSCs in the presence of NMDA was significantly larger in 7/21 Purkinje cells. To summarize, the mean amplitude was 11.6±0.1 pA in control and 13.3±0.7 pA in the presence of NMDA (Figure 4, left).

Based on the observation that in a majority of Purkinje cell the amplitude of mEPSC is not changed by NMDA, we can proposed that the effect of NMDA is probably presynaptic, however we can not exclude any other more complicated effect.

**Figure 5:**
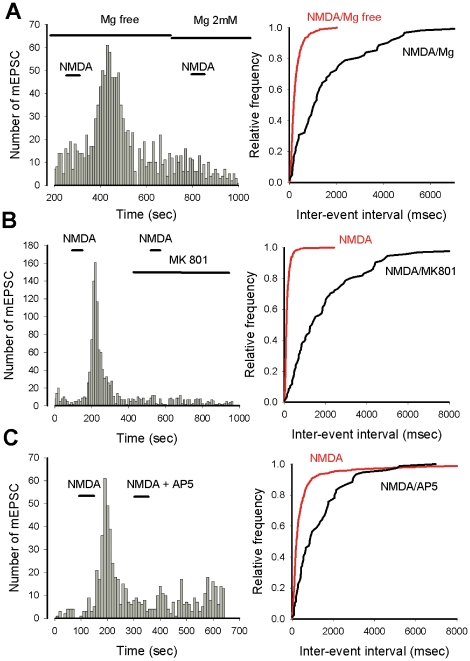
NMDA binds a NMDA receptor and increases mEPSC frequency. A. The NMDA effect on mEPSC frequency is blocked in the presence of external Mg^2+^. Histogram on the left represents the number of mEPSCs as a function of recording time (bin 10 s), NMDA is applied as indicated by the two short horizontal bars, in the absence or presence of external Mg^2+^ (as indicated by the two horizontal long bars). Right: cumulative plot of the inter-event intervals when NMDA is applied in the absence (red curve) or presence (black curve) of Mg^2+^. B and C. The facilitatory effet of NMDA on mEPSC frequency is blocked by two NMDA receptor antagonists, MK801 and AP5 respectively. Left, histograms represent the number of mEPSCs as a function of recording time (bin 10 s). NMDA and NMDA receptor antagonists are applied as indicated by the horizontal bars. Right, cumulative plots of inter-event intervals in the presence of NMDA alone (red curves), or in the presence of receptor antagonists (black curves). Note that in the presence of Mg^2+^, MK801 and AP5, the distributions of inter-event intervals are shifted towards longer intervals.

The next series of experiments illustrated in Fig. 5 were performed to determine if the effect of NMDA on mEPSC frequency was produced via the activation of NMDA receptors. As illustrated in figure 5A the effect of NMDA on mEPSC frequency was almost abolished (p<0.0001) when NMDA was applied in the presence of 2 mM extracellular Mg^2+^ (n = 3) or in the presence of NMDA receptor antagonists such as MK801 (Fig. 3B, n = 3) and AP5 (Fig. 5C, n = 3). These experiments demonstrate that NMDA exerts its effect on mEPSC frequency via activation of a pharmacologically identified NMDA receptor sensitive to external Mg^2+^.

**Figure 6:**
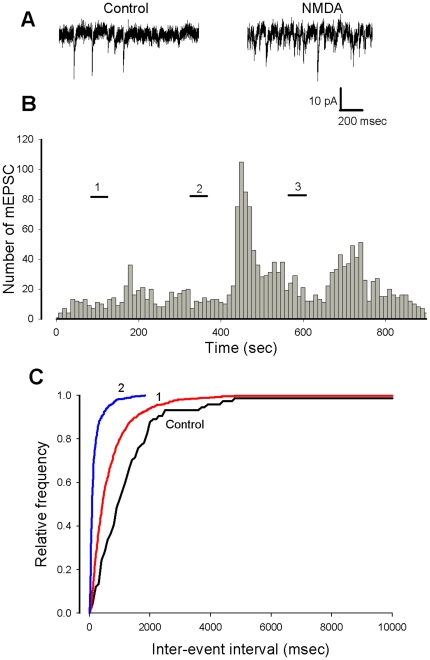
Repetitive applications of NMDA promote the facilitatory effect on mEPSC frequency. A. Current traces showing mEPSCs before (left) and after (right) a second application of NMDA. B (same cell as in A). Histogram representing the number of mEPSCs as a function of recording time (bin 10 s). During the short horizontal bars NMDA is applied at 10^−5^ M (bars 1 and 2) and at 5×10^−6^ M. (bar 3). C. Cumulative plots of inter-event intervals in control conditions (black curve) in the presence of NMDA after the first application (1, red curves) and second application (2, blue curve).

In all cases (n = 4 out of 4), the NMDA dependent increase in mEPSC frequency was significantly promoted (p<0.005) at the second application of the agonist (Fig. 6). Furthermore the effect of NMDA was detected at a concentration of 5×10^−6^ M. (Fig. 6).

The presynaptic effect of NMDA on mEPSCs is also observed in acute slices prepared from juvenile mice but requires a specific protocol of application.

The use of cerebellar organotypic slice cultures clearly shows an age dependent facilitatory effect of NMDA on spontaneous glutamate release by granule cells on Purkinje cells. However, to exclude the possibility that this effect of NMDA is due to modifications of native properties on neurons introduced by the culture system, we also investigated the effect of NMDA on isolated mEPSCs recorded in Purkinje cells from acute slices prepared from juvenile (P14–17) or adult (P40–50) mice.

**Figure 7:**
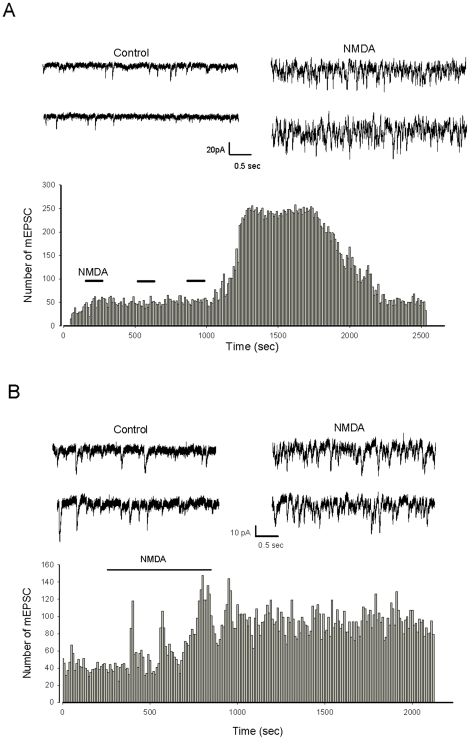
NMDA increases the frequency of mEPSCs in Purkinje cells recorded in acute slices prepared from P15–16 mice. A, B. Upper panels illustrate current traces recorded at −60 mV in the presence of TTX and Gabazine before (left) and after NMDA application (right). Lower panels show histograms representing the number of mEPSC as a function of recording time (bin 10 s). NMDA 10^−4^ M is applied as indicated by the horizontal bars. Note in A that the NMDA effect on mEPSC frequency is observed after the third application of NMDA, whereas in B the response is observed after a long-lasting (10 minute) application of NMDA.

**Figure 8:**
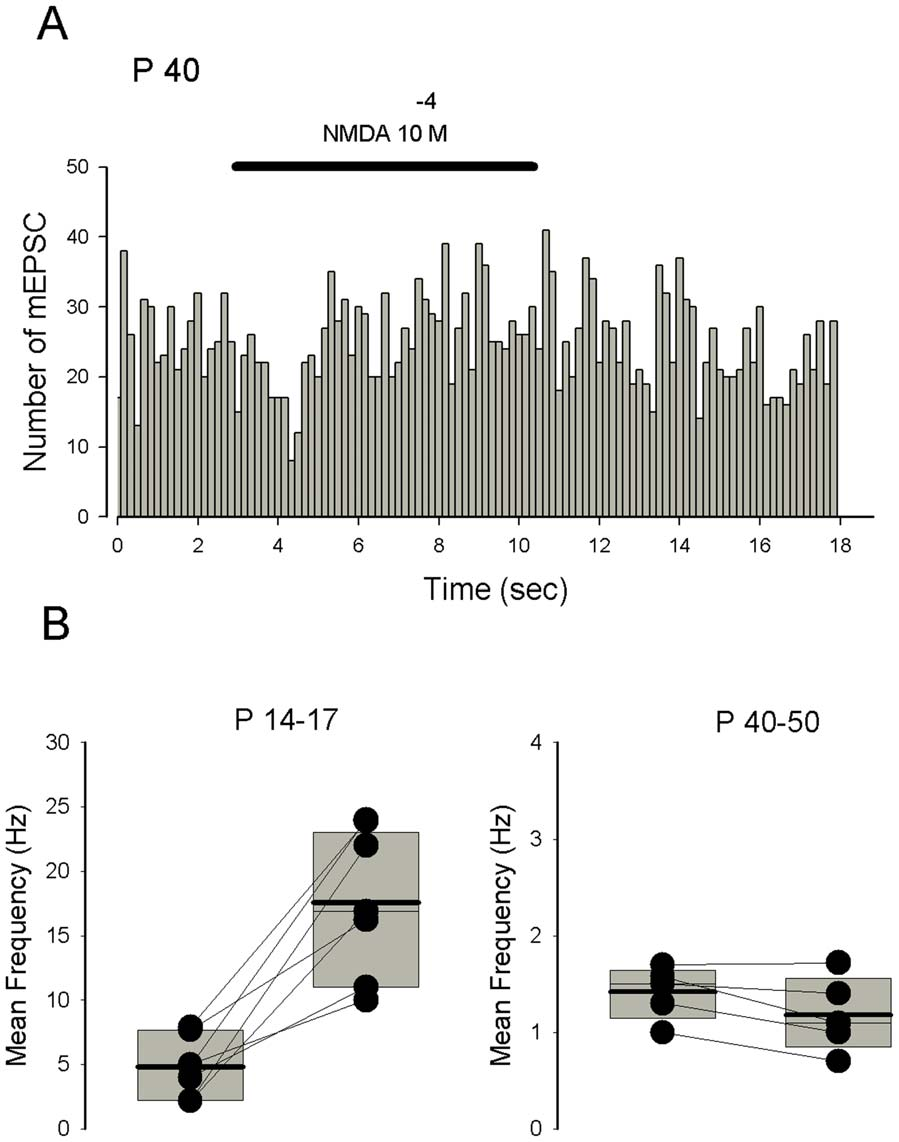
The effect of NMDA on mEPSC frequency is seen in juvenile but not adult mice. A. Histogram representing the number of mEPSCs recorded in a Purkinje cell from a slice prepared from a P40 mouse as a function of recording time (bin 10 s). During the horizontal bars NMDA is applied at 5×10^−4^ Note the absence of NMDA effect on mEPSC frequency. B. The effect of NMDA on mEPSC mean frequency recorded in Purkinje cell from acute slices prepared with juvenile (left, 7 cells) and adult mice (right, 5 cells). A representation with box plots indicating the mean values (thick line) and median values (thin line) has been adopted because of the non-normal distribution of frequency values. The bottom and the top of the box represent the 25^th^ and 75 th percentile respectively.

Interestingly, in juvenile mice whereas NMDA (10^−4^ M) applied for 2 minutes did not modify significantly the frequency of mEPSCs (n = 4), a facilitatory effect on mEPSC frequency was revealed after three successive applications of NMDA with an interval of 2 minutes (Fig. 7A, n = 3) or after a single long lasting (at least 10 minutes) application of the agonist (Fig. 7B, n = 4). The age-dependent presynaptic effect of NMDA on glutamate release was confirmed using acute slices. No effect of long duration applications of NMDA on mEPSC frequency was observed in Purkinje cells recorded from adult mice (P40–50, n = 5, Fig. 8A).

Fig. 8B summarizes the effects of NMDA on mEPSC frequency recorded in Purkinje cells from juvenile (left) and adult (right) mice. In juvenile mice the frequency of mEPSCs increased significantly from 4.8±0.3 Hz to 17.±2.1 Hz in the presence of NMDA (n = 4, ± SE, .P = 0.0013), whereas in adult mice the frequency was almost unchanged, (1.4±0.3 Hz in control and 1.2±0.2 Hz in the presence of NMDA, n = 5±SE)

## Discussion

Our data using cerebellar slice cultures and acute slices prepared from mice suggest that NMDA facilitates glutamate release onto Purkinje cells via a presynaptic effect in young (17–22 day old) slice cultures and in juvenile mice, and activates extrasynaptic receptors in old (age >28 day old) Purkinje cells from slice cultures.

### Postsynaptic and extrasynapticNMDA receptors in Purkinje cells

Many controversies exist concerning the presence of functional NMDA receptors in rodent Purkinje cells. Twenty years ago NMDA receptors were detected in rat Purkinje cells [33], [34], [35] but it was suggested they there were transiently expressed during a short window of time with a peak around P5 [34]. In agreement, whereas continuous expression of NR1 was seen in rat Purkinje cells, transient expression of mRNA for NR2D was observed during the first eight postnatal days [7]. Furthermore, in adult rats whereas NR1 proteins were detected, staining with NR2A/B antibody was low [8]. Finally, NMDA channels with characteristics of NR1/NR2D-containing NMDA receptors were recorded in Purkinje cells from young rats [16]. It was proposed that **in mice** NMDARs transiently produce membrane current in Purkinje cells that may serve as one of the epigenetic factors that support the survival of Purkinje cells [36]. Nevertheless, a NMDA component in the climbing fiber response was identified recently in adult (P25–P35) rat Purkinje cell using calcium imaging [19] whereas no NMDA receptor-mediated component was found in rat Purkinje cells as early as P0 [37]. To explain such discrepancies between the different studies, we can not exclude that several peaks of expression of NMDA receptors take place during development, depending on age and sensorial experience.

Interestingly, the pattern of expression of NMDA subunits was shown to be different between adult rats and mice [10]. Whereas adult Purkinje cells in mice were moderately stained for NR2A, rat Purkinje cells were immunonegative indicating species differences. Macroscopic NMDA currents and single NMDA channels were recorded in adult mice Purkinje cells indicating the presence of extrasynaptic NMDA receptors [17], [18] Furthermore, a NMDA component was also detected following climbing fiber stimulation, indicating the presence of synaptic NMDA receptors [17], [18] Interestingly, the NMDA component of the climbing fiber response appeared around P22 [17].

Our data on mice Purkinje cells recorded in slice cultures confirm the presence of functional extra-synaptic NMDA receptors in older Purkinje cells. The NMDA current we describe shares many properties with the NMDA current recorded after bath applications of NMDA in acute slices by Piochon et al. [17]. Indeed, the current is small, noisy, and insensitive to NBQX. The single channels we recorded are high-conducting channels indicating the presence of NR2A/B subunits [18]. Our data also demonstrate that the presence of climbing fibers is not required for the expression of functional extrasynaptic NMDA receptors in older mice Purkinje cells.

### Presynaptic effect of NMDA on glutamate release by granule cells

Presynaptic regulation of transmitter release is an efficient mechanism for providing feedback control in the CNS. In the cerebellum, a presynaptic facilitatory effect of NMDA was described at synapses between inhibitory interneurons and Purkinje cells. NMDA was able to increase the frequency of mIPSCs recorded in Purkinje cells [24]. NMDA also had a trophic effect on cerebellar interneuron GABAergic terminals and enhanced GABA release [26]. We found that NMDA also increases mEPSC frequency. An effect of NMDA on mEPSC frequency has not been detected in rat Purkinje cells recorded in acute slices [24], but such experiments were performed in the presence of 1 mM Mg^2+^. Nevertheless, a possible species difference can not be excluded.

The facilitatory effect of NMDA on mEPSCs could be mediated by calcium entry through NMDA receptor channels [38] and/or calcium channels activated by the subsequent depolarization produced by opening of NMDA receptor channels. The resulting increase of internal calcium may directly trigger the release or increase the efficacy of the release machinery ([2], for a review).

Facilitatory presynaptic effects of NMDA on glutamate release were initially demonstrated in the entorhinal cortex [39]. Interestingly, we show that the effect of NMDA on mEPSCs occurs only in young slice cultures or in acute slices prepared from juvenile mice. Evidence exists indicating that the function of presynaptic NMDA receptors decreases during development, as seen in hippocampus, enthorinal cortex and visual cortex ([2], for a review and references). In addition our data suggest that a switch between pre and post synaptic effects of NMDA on Purkinje cells could occur at around P23. Similarly, it has been proposed that in cerebellar interneurons the localization of AMPA receptors shifts from the axonal to the somato-dendritic compartment [40]. Furthermore, during development the glutamatergic modulation of GABA release in stellate cells switches from an AMPA-mediated transient inhibition to a NMDA-receptor-induced potentiation [24]. As already quoted for Purkinje cell extra-synaptic NMDA receptors, the presence of climbing fibers is not required for the developmental switch to occur. However, it has been shown that extra-synaptic NMDA receptors contain NR2B subunits while NR2A are mainly located at the climbing fiber synapses [18], suggesting that climbing fibers could play a role in determining differential subunit composition at the synaptic versus extra-synaptic sites.

Where are NMDA receptors responsible for the faciltatory effect on mEPSC frequency localized? They could be present at parallel fiber terminals or in another cell type. Recently the presence of NMDA receptors in parallel fibers (17–24 days old rats) was demonstrated by immunoelectron microscopy [31]. These receptors are essentially composed of NR1 and NR2A subunits. The effect of NMDA may also take place at granule cell dendrites rather than at parallel fiber terminals, but in this case the effect of NMDA should be observed in all young Purkinje cells recorded in slice cultures, independently of the age of culture or mouse donor.

Activation of presynaptic NMDA receptors has been shown to be required for long-term plasticity in diverse structures ([4], for a review). Many forms of synaptic plasticity have been identified at the parallel fiber-Purkinje cell synapses. Two forms of LTD required activation of NMDA receptors Presynaptic NMDA receptors localized at parallel fiber terminals have been proposed to be involved in LTD requiring high-frequency parallel fiber activity [28], [31], whereas postsynaptic NMDA receptors assume a key role in the LTD induced by paired parallel and climbing fiber activation in the mature mouse cerebellum [19]. Because the presynaptic effect of NMDA we describe here depends on the age of cultures or animals, it would be interesting to know if NMDA-dependent forms of synaptic plasticity at the parallel fiber-Purkinje cell synapses are regulated during development. In this context a late developmental switch toward NMDA receptor-dependent plasticity has been identified recently [19].

Several previous studies show that NMDA can have opposing effects on miniature and evoked synaptic currents, for example NMDA increases mIPSC frequency and decreases evoked IPSCs [24]. Thus the facilitatory effects of NMDA on mEPSC frequency we describe may participate in presynaptic LTD by preventing evoked release.

The observation that long lasting or repetitive applications of NMDA are required (particularly in acute slices) to elicit the presynaptic effect of NMDA on glutamate release suggests that second messengers may play a role, and/or that downstream pathways have to be activated.

## References

[1] MacDonald JF, Nowak LM (1990). Mechanisms of blockade of excitatory amino acid receptor channels.. Trends Pharmacol Sci.

[2] Corlew R, Brasier DJ, Feldman DE, Philpot BD (2008). Presynaptic NMDA receptors: newly appreciated roles in cortical synaptic function and plasticity.. Neuroscientist.

[3] Köhr G (2006). NMDA receptor function: subunit composition versus spatial distribution.. Cell Tissue Res.

[4] Duguid I, Sjöström PJ (2006). Novel presynaptic mechanisms for coincidence detection in synaptic plasticity.. Curr Opin Neurobiol.

[5] Yashiro K, Philpot BD (2008). Regulation of NMDA receptor subunit expression and its implications for LTD, LTP, and metaplasticity.. Neuropharmacol.

[6] Paoletti P, Neyton J (2007). NMDA receptor subunits: function and pharmacology.. Curr Opin Pharmacol.

[7] Akazawa C, Shigemoto R, Bessho Y, Nakanishi S, Mizuno N (1994). Differential expression of five N-methyl-D-aspartate receptor subunit mRNAs in the cerebellum of developing and adult rats.. J Comp Neurol.

[8] Petralia RS, Wang YX, Wenthold RJ (1994). The NMDA receptor subunits NR2A and NR2B show histological and ultrastructural localization patterns similar to those of NR1.. J Neurosci.

[9] Cathala L, Misra C, Cull-Candy S (2000). Developmental profile of the changing properties of NMDA receptors at cerebellar mossy fiber-granule cell synapses.. J Neurosci.

[10] Thompson CL, Drewery DL, Atkins HD, Stephenson FA, Chazot PL (2000). Immunohistochemical localization of N-methyl-D-aspartate receptor NR1, NR2A, NR2B and NR2C/D subunits in the adult mammalian cerebellum.. Neurosci Lett.

[11] Iijima K, Abe H, Okazawa M, Moriyoshi K, Nakanishi S (2008). Dual regulation of NR2B and NR2C expression by NMDA receptor activation in mouse cerebellar granule cell cultures.. Proc Natl Acad Sci U S A.

[12] Wee KS, Zhang Y, Khanna S, Low CM (2008). Immunolocalization of NMDA receptor subunit NR3B in selected structures in the rat forebrain, cerebellum, and lumbar spinal cord.. J Comp Neurol.

[13] D'Angelo E, De Filippi G, Rossi P, Taglietti V (1995). Synaptic excitation of individual rat cerebellar granule cells in situ: evidence for the role of NMDA receptors.. J Physiol.

[14] Billups D, Liu YB, Birnstiel S, Slater NT (2002). NMDA receptor-mediated currents in rat cerebellar granule and unipolar brush cells.. J Neurophysiol.

[15] Dupont JL, Fourcaudot E, Beekenkamp H, Poulain B, Bossu JL (2006). Synaptic organization of the mouse cerebellar cortex in organotypic slice cultures.. Cerebellum.

[16] Misra C, Brickley SG, Farrant M, Cull-Candy SG (2000). Identification of subunits contributing to synaptic and extrasynaptic NMDA receptors in Golgi cells of the rat cerebellum.. J Physiol (Lond).

[17] Piochon C, Irinopoulou T, Brusciano D, Bailly Y, Mariani J (2007). NMDA receptor contribution to the climbing fiber response in the adult mouse Purkinje cell.. J Neurosci.

[18] Renzi M, Farrant M, Cull-Candy SG (2007). Climbing-fibre activation of NMDA receptors in Purkinje cells of adult mice.. J Physiol.

[19] Piochon C, Levenes C, Ohtsuki G, Hansel C (2010). Purkinje cell NMDA receptors assume a key role in synaptic gain control in the mature cerebellum.. Neurosci.

[20] Howe JR, Cull-Candy SG, Colquhoun D (1991). Currents through single glutamate receptor channels in outside-out patches from rat cerebellar granule cells.. J Physiol.

[21] Carter AG, Regehr WG (2000). Prolonged synaptic currents and glutamate spillover at the parallel fiber to stellate cell synapse.. J Neurosci.

[22] Brickley SG, Misra C, Mok MH, Mishina M, Cull-Candy SG (2003). NR2B and NR2D subunits coassemble in cerebellar Golgi cells to form a distinct NMDA receptor subtype restricted to extrasynaptic sites.. J Neurosci.

[23] Misra C, Brickley SG, Wyllie DJ, Cull-Candy SG (2000). Slow deactivation kinetics of NMDA receptors containing NR1 and NR2D subunits in rat cerebellar Purkinje cells.. J Physiol.

[24] Glitsch M, Marty A (1999). Presynaptic effects of NMDA in cerebellar Purkinje cells and interneurons.. J Neurosci.

[25] Liu SJ (2007). Biphasic modulation of GABA release from stellate cells by glutamatergic receptor subtypes.. J Neurophysiol.

[26] Fiszman ML, Barberis A, Lu C, Fu Z, Erdélyi F (2005). NMDA receptors increase the size of GABAergic terminals and enhance GABA release.. J Neurosci.

[27] Casado M, Dieudonné S, Ascher P (2000). Presynaptic N-methyl-D-aspartate receptors at the parallel fiber-Purkinje cell synapse.. Proc Natl Acad Sci U S A.

[28] Casado M, Isope P, Ascher P (2002). Involvement of presynaptic N-methyl-D-aspartate receptors in cerebellar long-term depression.. Neuron.

[29] Qiu DL, Knöpfel T (2009). Presynaptically expressed long-term depression at cerebellar parallel fiber synapses.. Pflugers Arch.

[30] Qiu DL, Knöpfel T (2007). An NMDA receptor/nitric oxide cascade in presynaptic parallel fiber-Purkinje neuron long-term potentiation.. J Neurosci.

[31] Bidoret C, Ayon A, Barbour B, Casado M (2009). Presynaptic NR2A-containing NMDA receptors implement a high-pass filter synaptic plasticity rule.. Proc Natl Acad Sci U S A.

[32] Gähwiler BH (1981). Organotypic monolayer cultures of nervous tissue.. J Neurosci Methods.

[33] Dupont JL, Gardette R, Crepel F (1987). Postnatal development of the chemosensitivity of rat cerebellar Purkinje cells to excitatory amino acids. An in vitro study.. Brain Res.

[34] Krupa M, Crepel F (1990). Transient Sensitivity of Rat Cerebellar Purkinje Cells to N-methyl-D-aspartate during Development. A Voltage Clamp Study in in vitro Slices.. Eur J Neurosci.

[35] Momiyama A, Feldmeyer D, Cull-Candy SG (1996). Identification of a native low-conductance NMDA channel with reduced sensitivity to Mg2+ in rat central neurones.. J Physiol.

[36] Yuzaki M, Forrest D, Verselis LM, Sun SC, Curran T (1996). Functional NMDA receptors are transiently active and support the survival of Purkinje cells in culture.. J Neurosci.

[37] Lachamp P, Balland B, Tell F, Baude A, Strube C, Crest M, Kessler JP (2005). Early expression of AMPA receptors and lack of NMDA receptors in developing rat climbing fibre synapses.. J Physiol.

[38] Glitsch MD (2008). Calcium influx through N-methyl-D-aspartate receptors triggers GABA release at interneuron-Purkinje cell synapse in rat cerebellum.. Neurosci.

[39] Berretta N, Jones RS (1996). Tonic facilitation of glutamate release by presynaptic N-methyl-D-aspartate autoreceptors in the entorhinal cortex.. Neurosci.

[40] Bureau I, Mulle C (1998). Potentiation of GABAergic synaptic transmission by AMPA receptors in mouse cerebellar stellate cells: changes during development.. J Physiol.

